# Radiomic prediction for durable response to high‐dose methotrexate‐based chemotherapy in primary central nervous system lymphoma

**DOI:** 10.1002/cam4.70182

**Published:** 2024-09-10

**Authors:** Haoyi Li, Mingming Xiong, Ming Li, Caixia Sun, Dao Zheng, Leilei Yuan, Qian Chen, Song Lin, Zhenyu Liu, Xiaohui Ren

**Affiliations:** ^1^ Department of Neurosurgery Beijing Tiantan Hospital, Capital Medical University Beijing China; ^2^ National Genomics Data Center Beijing Institute of Genomics, Chinese Academy of Sciences and China National Center for Bioinformation Beijing China; ^3^ CAS Key Laboratory of Molecular Imaging Beijing Key Laboratory of Molecular Imaging, Institute of Automation, Chinese Academy of Sciences Beijing China; ^4^ Department of Nuclear Medicine Beijing Tiantan Hospital, Capital Medical University Beijing China; ^5^ School of Artificial Intelligence, University of Chinese Academy of Sciences Beijing China

**Keywords:** methotrexate, primary central nervous system lymphoma, prognosis, radiomics, response

## Abstract

**Background:**

The rarity of primary central nervous system lymphoma (PCNSL) and treatment heterogeneity contributes to a lack of prognostic models for evaluating posttreatment remission. This study aimed to develop and validate radiomic‐based models to predict the durable response (DR) to high‐dose methotrexate (HD‐MTX)‐based chemotherapy in PCNSL patients.

**Methods:**

A total of 159 patients pathologically diagnosed with PCNSL between 2011 and 2021 across two institutions were enrolled. According to the NCCN guidelines, the DR was defined as the remission lasting ≥1 year after receiving HD‐MTX‐based chemotherapy. For each patient, a total of 1218 radiomic features were extracted from prebiopsy T1 contrast‐enhanced MR images. Multiple machine‐learning algorithms were utilized for feature selection and classification to build a radiomic signature. The radiomic‐clinical integrated models were developed using the random forest method. Model performance was externally validated to verify its clinical utility.

**Results:**

A total of 105 PCNSL patients were enrolled after excluding 54 cases with ineligibility. The training and validation cohorts comprised 76 and 29 individuals, respectively. Among them, 65 patients achieved DR. The radiomic signature, consisting of 8 selected features, demonstrated strong predictive performance, with area under the curves of 0.994 in training cohort and 0.913 in validation cohort. This signature was independently associated with the DR in both cohorts. Both the radiomic signature and integrated models significantly outperformed the clinical models in two cohorts. Decision curve analysis underscored the clinical utility of the established models.

**Conclusions:**

This radiomic signature and integrated models have the potential to accurately predict the DR to HD‐MTX‐based chemotherapy in PCNSL patients, providing valuable therapeutic insights.

## 1 INTRODUCTION

Primary central nervous system lymphoma (PCNSL) is a rare extranodal non‐Hodgkin lymphoma that only involves the brain, spine, cerebrospinal fluid, and eyes without evidence of systematic spread.^1^ Over the past decades, high‐dose methotrexate (HD‐MTX)‐based polychemotherapy regimens have been widely adopted as the standard care for PCNSL patients.^2^ Despite significant advancements in tumor control, the early recurrence rate remains high, resulting in 12.5%–60.0% of patients experiencing tumor progression within 1 year of completing initial treatment.^1^, ^3^, ^4^, ^5^, ^6^ Notably, only a quarter of the patients had a remission lasting over 3 years after receiving HD‐MTX treatment.^4^, ^7^ This observed discrepancy may derive from individual heterogeneity in the physiological response to MTX, compounded by a variety of adjuvant medications used across different institutions and regions.^2^ Nevertheless, the use of MTX as the backbone in PCNSL treatment remains undisputed.^2^, ^8^

National Comprehensive Cancer Network (NCCN) guidelines indicate that the absence of response to treatment is declared if a patient receiving HD‐MTX‐based chemotherapy without radiotherapy experienced remission less than 1 year.^9^ Evidence has shown that patients who completed chemotherapy but experienced tumor progression within 1 year had a dismal prognosis, with a median survival of only 2 months.^2^ In addition, administration of HD‐MTX has proven to have significant side effects, which may be challenging for elderly patients with reduced physiological reserves.^4^, ^10^ Therefore, early identification of the special population who receive HD‐MTX‐based chemotherapy with durable remission/response (≥1 year) is crucial. It will facilitate the development of individual therapeutic strategies and improve tumor remission rates.

Radiomics is a noninvasive technique that utilizes high‐throughput data‐mining algorithms to extract information from medical imaging, generating high‐dimensional, mineable and quantitative metrics that reflect the underlying pathophysiology and tumor heterogeneity characteristics.^11^, ^12^, ^13^ While radiomics has been widely employed in differentiating between PCNSL and glioblastoma,^14^, ^15^ the correlation between radiomic features and therapy response in PCNSL remains relatively unclear.^15^ Several studies have reported the prognostic potential of radiomic signatures in predicting outcomes in PCNSL patients, although validation from independent external groups has been lacking.^16^, ^17^ A recent study by Destito et al. employed radiomics‐based machining learning tools to predict overall and progression‐free survival in PCNSL.^18^ However, the selected radiomic features may not yet have a well‐established value for individual prognosis due to the complexities and inconsistencies in treatment details. Furthermore, given the absence of consensus regarding systematic chemotherapy regimens for PCNSL, current clinical models such as the International Extranodal Lymphoma Study Group (IELSG) score^19^ and Memorial Sloan‐Kettering Cancer Center (MSKCC) classification^20^ may not have optimal predictive performance for prognosis. Therefore, the imperative now lies in prioritizing the development of enhanced biologic and radiologic predictive models for PCNSL, which are crucial to propel therapeutic advancements.

The primary objectives of this study are as follows: (i) utilize machine learning‐based algorithms to identify the pivotal radiomic characteristics that could predict a durable response (DR) to HD‐MTX‐based chemotherapy (remission lasting more than 1 year after treatment) in PCNSL patients; (ii) develop integrated models that combined both radiomics and clinical features, aiming to improve the predictive capacity for DR to HD‐MTX‐based treatment in PCNSL patients; and (iii) conduct an external validation to assess the reliability and robustness of our findings using data from another institution.

## 2 MATERIALS AND METHODS

### 2.1 Study protocol approvals and patient consents

This retrospective study was approved by the Institutional Review Board of our institution (YW2019‐016‐11). Written informed consent for patient was waived.

### 2.2 Patient assessment and variable collection

**FIGURE 1:**
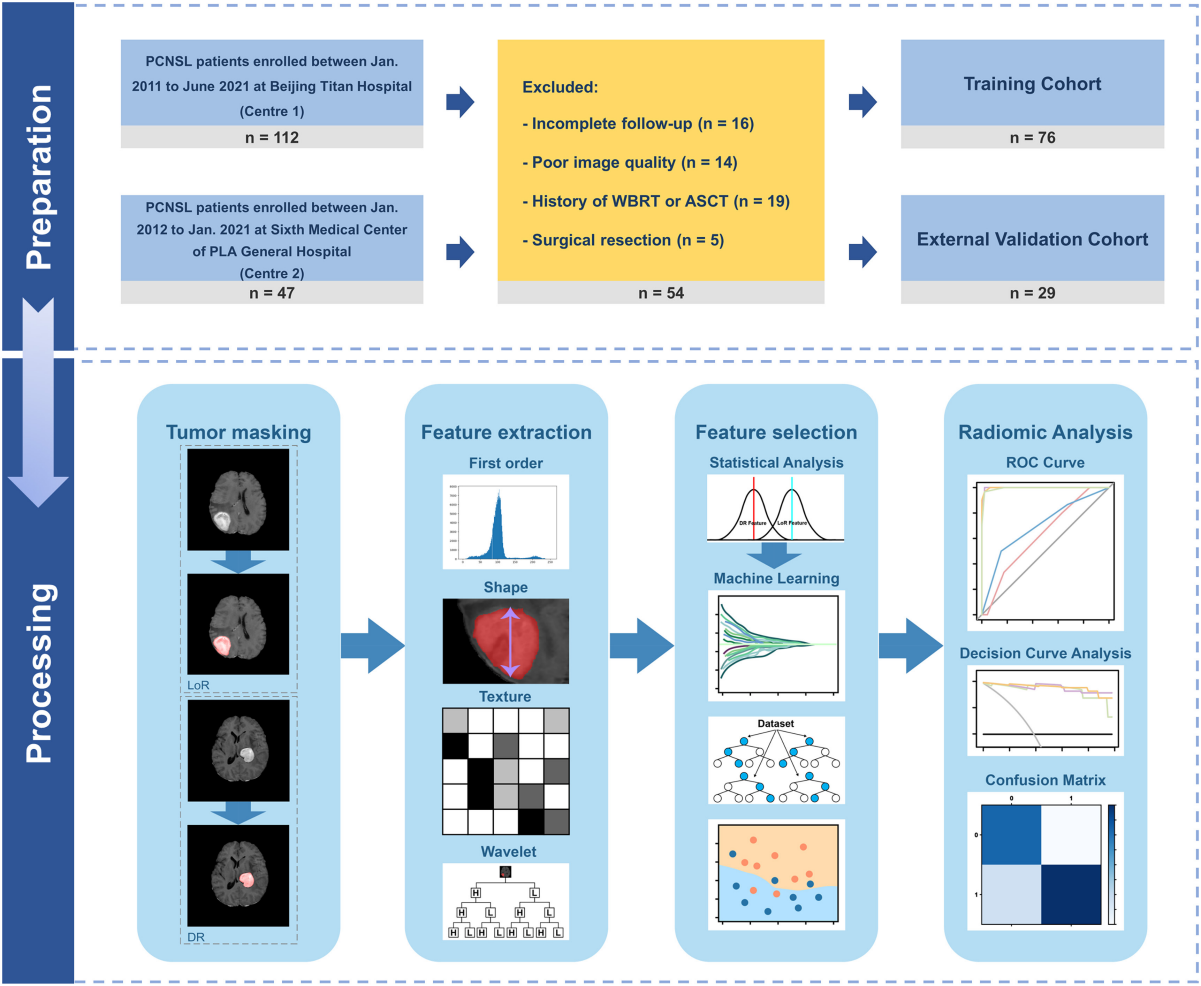
Flow diagrams of patient selection and radiomics construction. ASCT, autologous stem cell transplant; DR, durable response; LoR, loss of response; ROC, receiver operating characteristic; WBRT, whole brain radiotherapy.

This study retrospectively enrolled patients pathologically diagnosed with PCNSL between 2011 and 2021 based on the institutional databases of Beijing Tiantan Hospital (Centre 1 as the training cohort) (*n* = 112) and the Sixth Medical Center of PLA General Hospital (Centre 2 as the external validation cohort) (*n* = 47) (Figure 1). Biopsy‐proven PCNSL patients who were immunocompetent, 18 years of age or older, had normal liver and renal functions before chemotherapy, received neither whole brain radiotherapy (WBRT) nor autologous stem cell transplant (ASCT), and completed T1 contrast‐enhancement (T1CE) MR imaging with intact follow‐up information were eligible for enrollment in this study. Patients who had craniotomy due to PCNSL itself or other CNS diseases, administration of corticosteroids and/or Bruton tyrosine kinase (BTK) inhibitor therapy during the initial treatment period, evidence of extra‐CNS involvement, a low KPS score (≤50) unrelated to PCNSL, poor brain image quality, active infection, concomitant malignancy, pregnancy, or breastfeeding were excluded. Baseline clinical data included age, gender, Karnofsky performance scale (KPS) score, Eastern Cooperative Oncology Group‐Performance Status (ECOG‐PS), IELSG scores, MSKCC scores, tumor location, and treatment strategies.

### 2.3 Treatment protocol

The initial treatment consisted of an induction phase and a consolidation phase. During the induction period, each patient received 4–6 cycles of HD‐MTX (3 g/m^2^) intravenously on Day 1 of a 14‐day cycle. Among all patients, 61 individuals were administered HD‐MTX alone, and the remaining received HD‐MTX plus adjuvant therapy (details in Table 1). As recommended by NCCN guidelines, the chemotherapy regimens employed during the induction stage were to be maintained without modification and continued into the consolidation phase if tolerated. Consequently, during the consolidation period, more than half of the patients continued with the original HD‐MTX‐based chemotherapy regimens per month for up to 1 year, while approximately one‐third of the patients were switched to alternative chemotherapy regimens due to intolerance and toxicity associated with HD‐MTX administration during the treatment period (Table 1). Supportive therapy included intravenous administration of folinic acid every 6 h following 24 h of HD‐MTX administration, and granulocyte colony‐stimulating factor shots if needed. Complete response (CR) was defined as per the International PCNSL Collaborative Group (IPCG) criteria.^21^ All chemotherapy regimens were completed for all patients regardless of whether the CR was achieved earlier. No grade 4 toxic effects were reported during the period of therapy according to the WHO's 1996 classification.

**TABLE 1:** Characteristics of patients in the training and validation cohorts.

Clinical characteristics	Overall Cohort (*n* = 105)	Training cohort (*n* = 76)	Validation cohort (*n* = 29)	*p*‐value
Age, years, median (IQR)	56 (50–62)	56 (50–62)	58 (50–62)	0.951
Gender, *n* (%)				0.723
Female	50 (47.6)	37 (48.7)	13 (44.8)	
Male	55 (52.4)	39 (51.3)	16 (55.2)	
KPS Score, median (IQR)	70 (60–80)	70 (60–80)	70 (60–80)	0.825
ECOG Score, median (IQR)	2 (1–3)	2 (1–3)	2 (1–3)	0.906
IELSG Score, median (IQR)	2 (1–3)	2 (1–2)	2 (2–3)	0.247
MSKCC class				0.979
Class I	26 (24.8)	19 (25.0)	7 (24.1)	
Class II	49 (46.7)	35 (46.1)	14 (48.3)	
Class III	30 (28.6)	22 (28.9)	8 (27.6)	
Involvement of deep areas				0.327
Yes	76 (72.4)	53 (69.7)	23 (79.3)	
No	29 (27.6)	23 (30.3)	6 (20.7)	
Multiple lesions				
Yes	57 (54.3)	39 (51.3)	18 (62.1)	
No	48 (45.7)	37 (48.7)	11 (37.9)	
Treatment				
Induction regimen				0.383
MTX alone	61 (58.1)	41 (53.9)	20 (69.0)	
R‐MTX	4 (3.8)	3 (3.9)	1 (3.4)	
R‐MTX‐TMZ‐LNDA	17 (16.2)	13 (17.1)	4 (13.8)	
R‐MT	4 (3.8)	3 (3.9)	1 (3.4)	
R‐MTX‐A	15 (14.3)	14 (18.4)	1 (3.4)	
R‐M‐LNDA	4 (3.8)	2 (2.6)	2 (6.9)	
Consolidation regimen				0.257
MTX alone	49 (46.7)	31 (40.8)	18 (62.1)	
MTX + other chemotherapy	5 (4.8)	4 (5.3)	1 (3.4)	
Other chemotherapy	32 (30.5)	25 (32.9)	7 (24.1)	
None	19 (18.1)	16 (21.1)	3 (2.9)	
Durable response				0.638
Yes	65 (61.9)	46 (60.5)	19 (65.5)	
No	40 (38.1)	30 (39.5)	10 (34.5)	

Abbreviations: A, Ara‐C (Cytarabine); ECOG, Eastern Cooperative Oncology Group‐Performance Status; IELSG, International Extranodal Lymphoma Study Group; IQR, interquartile range; KPS, Karnofsky performance sacle; LNDA, lenalidomide; MSKCC, Memorial Sloan‐Kettering Cancer Center; MTX (M), methotrexate; n, number; R, Rituximab; TMZ (T), temozolomide.

### 2.4 Follow‐up and endpoint

Patients treated in two institutions were followed up at an interval of 3 months. Progression‐free survival (PFS) was defined as the interval from the date of diagnosis to the date of recurrence or the last follow‐up. According to the NCCN guidelines, the endpoint was defined as remission lasting ≥1 year after HD‐MTX‐based chemotherapy, namely DR to HD‐MTX‐based chemotherapy. Specifically, DR to HD‐MTX‐based chemotherapy was determined by either of two criteria: (i) patients, who achieved CR after initial treatment, remained in remission for 1 year or longer without signs of new lesion appearance on T1CE MR images; (ii) patients, who did not achieve CR after initial treatment, experienced radiological maintenance or continuous reduction in the volume of targeted lesions for over 1 year without signs of new lesion appearance on T1CE MR images. However, the patients who completed initial treatment but lacked durable remission (more than 1 year) were considered to have loss of durable response (LoDR) to treatment.

### 2.5 Image acquisition and preprocessing

As recommended by IPCG,^21^ all patients underwent T1CE MR imaging with DTPA‐Gd injections before biopsy at two centers. The detailed parameters of MR image acquisition are described in Table S1. All T1CE scans were retrieved from the Picture Archiving and Communication System for further image processing. Prebiopsy MRIs were analyzed by an experienced neurosurgeon (M.L with 8 years of clinical experience). All segmentation masks were confirmed by a senior radiologist (L.L.Y) and a senior neurosurgeon (X.H.R) simultaneously, both of whom had more than 15 years of clinical experience. Disagreements were resolved through consensus‐based discussion. Regions of interest (ROIs, consisting of enhanced tumor plus necrosis) were manually outlined using the T1CE images via the itk‐SNAP software (www.itksnap.org) (Figure 1).

### 2.6 Radiomic signature analysis

#### 2.6.1 Radiomic feature extraction

Each patient's MRI was preprocessed with N4 bias field correction. Each scan was normalized with *z*‐scores to obtain a standard normal distribution of imaging intensities and resampled to the same resolution of 1 × 1 × 1mm^3^ voxels. Radiomic feature extraction was conducted using PyRadiomics (v3.1.0; Python Software Foundation). For each patient, a total of 1218 imaging features were automatically extracted, covering 18 first‐order statistical features, 14 shape‐based features, 68 texture features, and 1118 wavelet features. The first‐order statistical features, shape‐based features, and texture features were extracted from the original image. Texture features included 22 gray level cooccurrence matrix, 16 gray level run length matrix, 16 gray level size zone matrix, and 14 gray level dependence matrix features. The wavelet features were extracted from the images and filtered by the wavelet transform and Laplacian of Gaussian. Features were normalized with *z*‐scores after extraction. The ComBat feature harmonization method was utilized to correct for the batch effect introduced by different acquisition protocol scanners.^22^

#### 2.6.2 Feature selection

To reduce the potential bias of results and overfitting, a coarse‐to‐fine approach was utilized to optimize the feature selection process.^23^ Initially, an analysis of variance (ANOVA) was used to address the impact of scanner effects, which are nonbiological variations produced by image acquisition settings. Next, univariate analysis was performed using the Mann–Whitney *U* and Pearson correlation coefficient to compare the differences between the DR group and the LoR group for each radiomic feature. According to the ascending order of the *p*‐value in each analysis, the repeated features of the top 5% in both analyses were selected for further analysis. Afterward, five classifiers were employed to select relevant features, including recursive feature elimination based on a support vector machine, least absolute shrinkage and selection operator, extremely randomized trees, random forest (RF), and ridge regression. The optimal radiomic features were ultimately identified based on their repeated appearance among all five methods (Table S2). This selection process was initially carried out on the training set and then validated on the validation set.

#### 2.6.3 Construction and validation of the radiomic signature

Based on the selected features, the RF model was used to develop a radiomic signature for DR prediction. Models were trained in the training cohort and validated in the external validation cohort. Afterward, radiomic scores for each patient were calculated in both cohorts. The prediction probabilities derived from the RF model were employed to create a radiomic score (Radscore) that integrated multiple radiomic features. To assess the quantitative prediction performance of the radiomic signature, receiver operating characteristic curves (ROCs) and confusion matrices were plotted. A confusion matrix, including accuracy (ACC), sensitivity, specificity, positive predictive value (PPV), and negative predictive value (NPV), was computed to further evaluate the models.

### 2.7 Construction and validation of clinical‐radiomic integrated model

Based on IELSG scores or MSKCC classification integrating demographic and biological information (e.g., age and Karnofsky Performance Status scores), clinical models were developed for DR prediction using ridge regression. To improve prediction ability, radiomic‐clinical integrated models, namely the Radscore combined with either the IELSG model or the MSKCC model, were established with the RF algorithm in the training cohort, respectively. The performance of the integrated models was evaluated in the validation cohorts using ROC curves and confusion matrices. The Delong test was performed to compare the performance among the integrated models, radiomic signature, and clinical models. Essential scripts for model development and the specific parameters were uploaded to GitHub (https://github.com/xiongmingming/PCNSL.git).

### 2.8 Clinical use

Decision curve analysis (DCA) was plotted to evaluate the clinical net benefits of clinical models, radiomic signatures, and clinical‐radiomic integrated models at different threshold probabilities.^24^ Furthermore, the net reclassification index (NRI) and integrated discrimination improvement (IDI) were calculated to compare the clinical usefulness among the established models.

### 2.9 Statistical analysis

Categorical variables are expressed as percentages, while continuous variables are presented as medians with interquartile ranges (IQRs). Clinical data were compared between the DR and LoDR groups, as well as between the training and validation cohorts using the Mann–Whitney *U* test, chi‐square test, or Fisher's exact test, as appropriate. Generalized multivariable logistic analysis was conducted to identify durable response‐related favorable factors. Multivariable Cox regression model was employed to further investigated the correlation between radiomic signature and PFS. All statistical computations were performed using IBM SPSS v26.0 and Python v3.1.0. A *p* < 0.05 for two‐tailed tests was considered statistically significant.

## 3 RESULTS

### 3.1 Patient characteristics

As shown in Figure 1, a total of 159 PCNSL patients who received HD‐MTX‐based chemotherapy were initially included in the study. Of these, 112 patients were assigned to the training cohort, and 47 patients were assigned to the external validation cohort. Based on the inclusion and exclusion criteria, 54 patients were deemed ineligible and subsequently excluded from the analysis for the following reasons: 11 patients lacked follow‐up information, 14 patients had poor image quality, 19 patients underwent WBRT or ASCT, and 5 patients had surgical resections.

After careful screening, a total of 105 PCNSL patients were eligibly enrolled in this analysis (*n* [training cohort] = 76; *n* [validation cohort] = 29). The median follow‐up time of this cohort was 16 months with an IQR of 6–34 months. Among all eligible individuals, 65 patients had DR to HD‐MTX‐based chemotherapy. The baseline features of the patients are shown in Table 1 and Table S3. There were balanced baseline clinical parameters and no significant DR rate between the training cohort and validation cohort (61.9% for the training cohort vs. 60.5% for the validation cohort, *p* = 0.638). In the training cohort, several clinical characteristics were significantly different between the DR and LoDR groups, including age, KPS score, ECOG score, IELSG score, MSKCC classification, and treatment strategies. However, in the validation cohort, no significant differences were observed in clinical variables between the two groups.

### 3.2 Feature selection and radiomic signature construction and validation

According to the coarse‐to‐fine feature selection strategy, eight imaging features were finally selected from the T1CE sequence (Table S4). Based on these selected features, a radiomic signature was constructed using the RF model in the training cohort.

**FIGURE 2:**
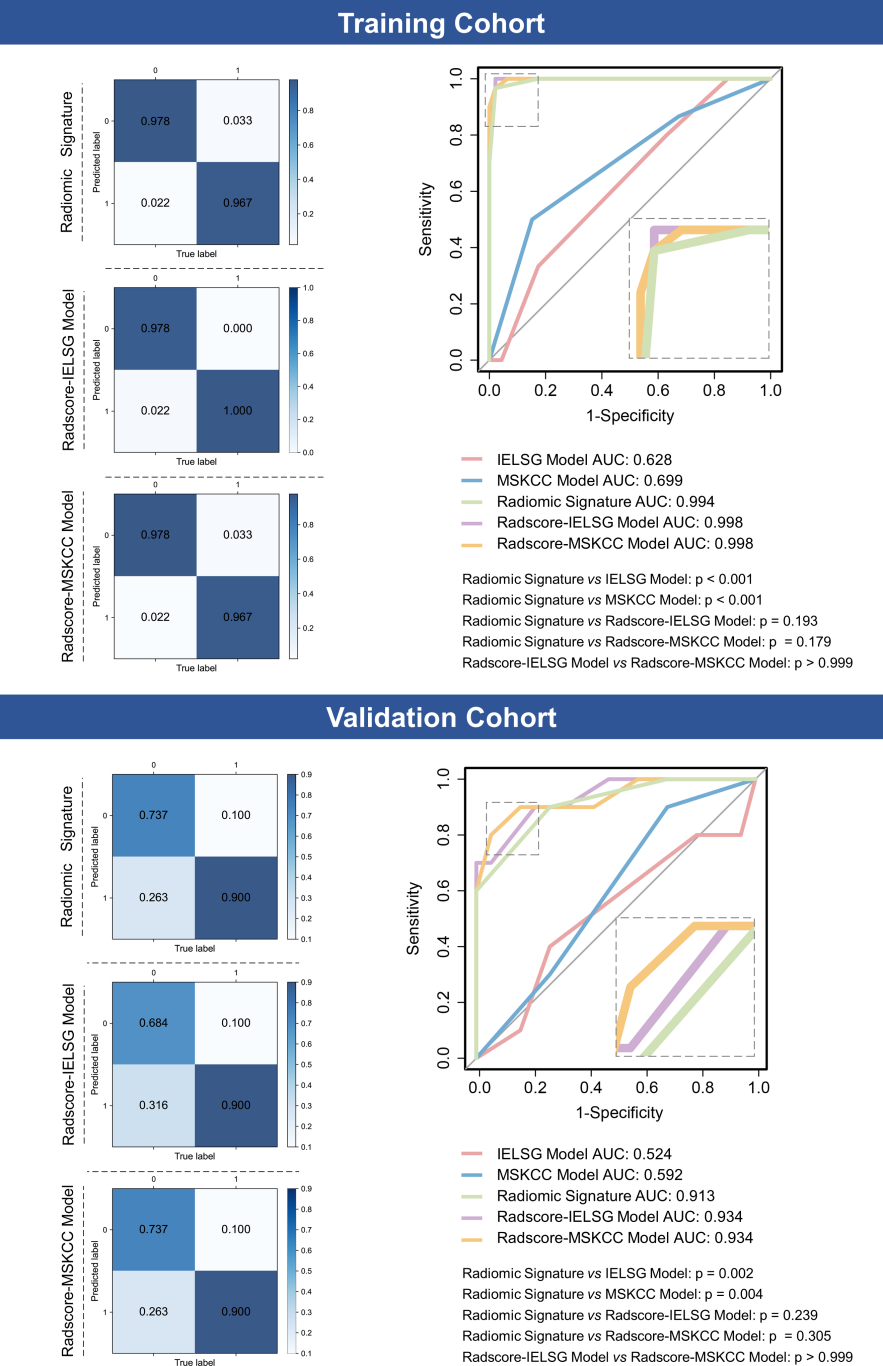
Predictive performance of the established models for differentiating response to high‐dose methotrexate‐based chemotherapy in the training and validation cohorts. For confusion matrices, the color depends on the number inside the square: The higher the number is, the darker the color. For receiver operating characteristic curves, the area under the curves were shown and compared among different models. AUC, area under the curve; IELSG, International Extranodal Lymphoma Study Group; MSKCC, Memorial Sloan‐Kettering Cancer Center.

There was a significant difference in the Radscore between the DR and LoDR groups in both cohorts (Table S3). In the training cohort, the radiomic signature yielded an impressive AUC of 0.994 (95% CI, 0.981–1.000), with an accuracy of 0.974, sensitivity of 0.967, specificity of 0.978, PPV of 0.967, and NPV of 0.978, for differentiating the DR and LoDR groups. In the validation cohort, the results were similar to those of the training cohort, with an AUC of 0.913, sensitivity of 0.900, and NPV of 0.933. However, it is important to note that the model performance of the validation cohort exhibited a decrease in accuracy (0.793), specificity (0.737), and PPV (0.643) when compared to those of the training cohort (Figure 2 and Table 3).

### 3.3 Association of radiomic signature with DR and PFS

To minimize confounding and prediction bias, a generalized multivariable logistic model was employed to investigate the independent association between the radiomic signature and DR. The analysis unveiled the independent predictive potential of the radiomic signature for distinguishing between the DR and LoDR groups. (beta, 1.210 SE, 0.216; *p* < 0.001) (Table 2). Consistently, this predictive capacity was observed after adjusting for confounders in the validation cohort (beta, 1.951 SE, 0.375; *p* < 0.001) (Table 2). We further investigated the impact of the radiomic signature on PFS. Multivariable Cox regression demonstrated that higher Radscore values were significantly associated with a decreased risk of relapse in both the training cohort (HR 0.005, 95% CI: 0.001–0.030, *p* < 0.001) and validation cohort (HR 8.3e^−5^, 95% CI: 6.1e^−7^−0.011, *p* < 0.001). Additionally, older age was found to contribute to an increased risk of recurrence (HR 1.060, 95% CI: 1.001–1.125, *p* = 0.045) in the training cohort. However, this significant correlation was not observed in the validation cohort (Table 3).

**TABLE 2:** Associations between covariables and durable response to treatment by generalized multivariable logistic regression.

	Training cohort				Validation cohort			
	beta	SE	*t*‐value	*p*‐value	beta	SE	*t*‐value	*p*‐value
Age	−0.008	0.004	−1.850	0.069	−0.010	0.009	−1.086	0.291
Gender (m/f)	−0.040	0.055	−0.730	0.468	−0.153	0.156	−0.984	0.337
ECOG Score	−0.026	0.037	−0.693	0.491	0.120	0.112	1.070	0.297
Deep lesions (y/n)	−0.036	0.080	−0.446	0.657	0.198	0.204	0.971	0.343
Multiple lesions (y/n)	−0.028	0.062	−0.458	0.648	−0.236	0.166	−1.416	0.172
IELSG Score	0.001	0.041	0.020	0.984	−0.006	0.095	−0.063	0.951
MSKCC Score	−0.067	0.064	−1.045	0.300	−0.256	0.170	−1.510	0.147
Radscore	1.210	0.216	15.223	<**0.001**	1.951	0.375	5.205	<**0.001**

*Note*: Boldface type indicates statistical significance with two‐sided *p* < 0.05.

Abbreviations: ECOG, Eastern Cooperative Oncology Group‐Performance Status; f, female; IELSG, International Extranodal Lymphoma Study Group; MSKCC, Memorial Sloan‐Kettering Cancer Center; MTX, methotrexate; n, no; SE, standard error; y, yes.

**TABLE 3:** Associations between covariables and recurrence by multivariable Cox regression.

	Training cohort			Validation cohort		
	HR	95% CI	*p*‐value	HR	95% CI	*p*‐value
Age	1.060	1.001–1.124	**0.045**	1.044	0.941–1.158	0.419
Gender (m/f)	0.688	0.343–1.381	0.293	0.754	0.174–3.259	0.754
ECOG Score	1.601	0.863–2.970	0.136	1.122	0.426–2.959	0.816
Deep lesions (y/n)	2.916	0.940–9.049	0.064	3.086	0.504–18.870	0.222
Multiple lesions (y/n)	1.554	0.683–3.536	0.293	3.371	0.031–22.222	0.146
IELSG Score	0.747	0.424–1.315	0.312	0.918	0.380–2.218	0.849
MSKCC Score	1.609	0.663–3.903	0.293	1.825	0.499–6.672	0.363
Radscore	0.005	0.001–0.030	<**0.001**	8.3e^−5^	6.1e^−7^−0.011	<**0.001**

*Note*: Boldface type indicates statistical significance with two‐sided *p* < 0.05.

Abbreviations: CI, confidential interval; ECOG, Eastern Cooperative Oncology Group‐Performance Status; f, female; HR, hazard ratio; IELSG, International Extranodal Lymphoma Study Group; MSKCC, Memorial Sloan‐Kettering Cancer Center; MTX, methotrexate; n, no; SE, standard error; y, yes.

### 3.4 Development and validation of the clinical‐radiomic integrated models

Based on the current clinical prognostic models, two integrated models, namely the Radscore‐IELSG and Radscore‐MSKCC models, were constructed. In the training cohort, these two integrated models achieved remarkable performance for differentiating the DR and LoDR groups, with AUCs of 0.998 (95% CI, 0.992–1.000) in the Radscore‐IELSG model and 0.998 (95% CI, 0.998–1.000) in the Radscore‐MSKCC model. For the Radscore‐ILESG model, confusion matrix analysis revealed outstanding accuracy, sensitivity, specificity, PPV, and NPV—0.987, 1.000, 0.978, 0.968, and 1.000, respectively. Similarly, the MSKCC‐Radscore model exhibited high accuracy, sensitivity, specificity, PPV, and NPV values of 0.974, 0.967, 0.967 and 0.978, respectively, facilitating discrimination between the DR and LoDR groups (Figure 2 and Table 4). In the validation cohort, these two integrated models maintained strong predictive performance, with an AUC of 0.934 for both the Radscore‐ILESG and MSKCC‐Radscore models. However, it is worth noting that the values of accuracy, specificity and PPV were reduced in the validation cohort compared to those in the training cohort (Figure 2 and Table 4).

**TABLE 4:** Performance of the established models.

	Training cohort					Validation cohort				
	IELSG model	MSKCC model	Radiomic signature	Radscore‐IELSG model	Radscore‐MSKCC model	IELSG model	MSKCC model	Radiomic signature	Radscore‐IELSG model	Radscore‐MSKCC model
Accuracy (95% CI)	0.632 (0.526–0.737)	0.711 (0.618–0.803)	0.974 (0.934–1.000)	0.987 (0.961–1.000)	0.974 (0.934–1.000)	0.586 (0.414–0.759)	0.586 (0.414–0.759)	0.793 (0.621–0.931)	0.759 (0.586–0.897)	0.793 (0.621–0.931)
AUC (95% CI)	0.628 (0.513–0.746)	0.699 (0.589–0.804)	0.994 (0.981–1.000)	0.998 (0.992–1.000)	0.998 (0.992–1.000)	0.524 (0.310–0.773)	0.592 (0.386–0.771)	0.913 (0.779–0.992)	0.934 (0.819–1.000)	0.934 (0.811–1.000)
Sensitivity (95% CI)	0.333 (0.171–0.517)	0.5 (0.321–0.667)	0.967 (0.897–1.000)	1.000 (1.000, 1.000)	0.967 (0.880, 1.000)	0.100 (0.0–0.334)	0.300 (0.000–0.615)	0.900 (0.667–1.000)	0.900 (0.667–1.000)	0.900 (0.667–1.000)
Specificity (95% CI)	0.826 (0.717–0.933)	0.848 (0.744–0.952)	0.978 (0.929–1.000)	0.978 (0.927–1.000)	0.978 (0.925–1.000)	0.842 (0.667–1.000)	0.737 (0.522–0.929)	0.737 (0.521–0.905)	0.684 (0.471–0.889)	0.737 (0.526–0.938)
PPV (95% CI)	0.556 (0.313, 0.800)	0.682 (0.476–0.889)	0.967 (0.893–1.000)	0.968 (0.892–1.000)	0.967 (0.889–1.000)	0.250 (0.000–0.890)	0.375 (0.000, 0.750)	0.643 (0.385–0.875)	0.600 (0.333–0.857)	0.643 (0.364–0.909)
NPV (95% CI)	0.655 (0.540–0.778)	0.722 (0.604–0.828)	0.978 (0.930–1.000)	1.000 (1.000, 1.000)	0.978 (0.929–1.000)	0.640 (0.440–0.826)	0.667 (0.444–0.857)	0.933 (0.769–1.000)	0.929 (0.750, 1.000)	0.933 (0.778–1.000)

Abbreviations: AUC, area under the curve; CI, confidential interval; IELSG, International Extranodal Lymphoma Study Group; MSKCC, Memorial Sloan‐Kettering Cancer Center; NPV, negative predictive value; PPV, positive predictive value.

### 3.5 Comparison of performance among established models

As illustrated in Figure 2, the predictive performance of the radiomic signature was superior to both the ILESG model (AUC: 0.994 vs. 0.628, *p* < 0.001) and the MSKCC model (AUC: 0.994 vs. 0.699, *p* < 0.001). Both clinical‐radiomic integrated models also significantly outperformed these two clinical models. However, the predictive ability was not significantly improved in either the Radscore‐ILESG model (AUC: 0.998 vs. 0.994, *p* = 0.193) or Radscore‐MSKCC model (AUC: 0.998 vs. 0.994, *p* = 0.179) compared to the radiomic signature. Notably, there was no significant difference in predictive performance between the two integrated models (*p* > 0.999). Similar comparative results were also found in the validation cohort.

### 3.6 Clinical use

**FIGURE 3:**
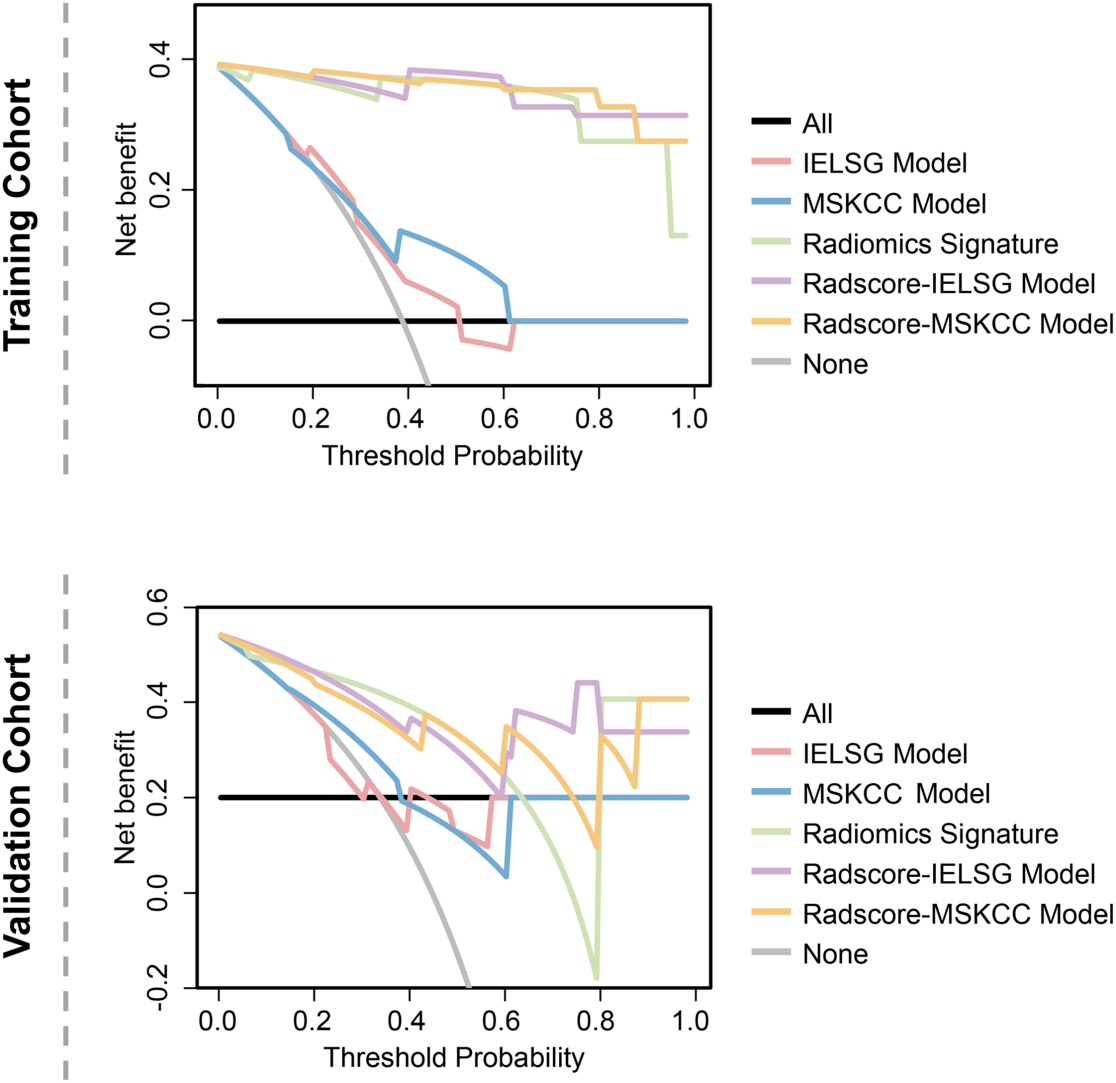
Decision curve analysis of the different prediction models in the training and validation cohorts. The black line represents the assumption that all patients have a durable response to high‐dose methotrexate‐based chemotherapy. The gray line represents the assumption that no patients have a durable response to high‐dose methotrexate‐based chemotherapy. Other different colored lines represent the different established models. The one with the highest net benefit at any given threshold is the preferred strategy. IELSG, International Extranodal Lymphoma Study Group; MSKCC, Memorial Sloan‐Kettering Cancer Center.

DCA showed that using the aforementioned established models to predict MTX‐treated DR added more benefit than either the treat‐all or treat‐none schemes in both the training and validation cohorts (Figure 3). Notably, as observed visually, the utilization of the Radscore‐ILESG model in clinical decision‐making may yield greater advantages compared to other established models in both training and validation cohorts (Figure 3). Furthermore, the NRI and IDI values consistently indicated that the radiomic signature exhibited enhanced predictive power in contrast to the two clinical models. Additionally, both integrated models might be particularly beneficial for clinical use when compared to the radiomic signature (IDI for the Radscore‐IELSG model: 0.08, IDI for the Radscore‐MSKCC model: 0.09; all *p* < 0.001) in the training cohort. However, there were no significant differences in the NRI and IDI values between the radiomic signature and the two integrated models in the validation cohorts (Table 5).

**TABLE 5:** Comparison of clinical use among the established models.

	Training cohort				Validation cohort			
	NRI		IDI		NRI		IDI	
	Value (95% CI)	*p*‐value	Value (95% CI)	*p*‐value	Value (95% CI)	*p*‐value	Value (95% CI)	*p*‐value
Radiomic signature vs IELSG model	0.79 (0.58–1.00)	<**0.001**	0.74 (0.67–0.82)	<**0.001**	0.69 (0.34–1.05)	<**0.001**	0.74 (0.67–0.82)	<**0.001**
Radiomic signature vs IELSG model	0.60 (0.37–0.83)	<**0.001**	0.67 (0.57–0.76)	<**0.001**	0.60 (0.18–1.02)	**0.005**	0.55 (0.28–0.82)	<**0.001**
Radscore‐IELSG model vs radiomic signature	0.03 (−0.03 to 0.10)	0.309	0.08 (0.04–0.12)	<**0.001**	−0.05 (−0.15 to 0.05)	0.304	−0.07 (−0.22–0.08)	0.371
Radscore‐MSKCC model vs radiomic signature	0.00 (0.00–0.00)	–	0.09 (0.06–0.12)	<**0.001**	0.00 (0.00–0.00)	–	−0.03 (−0.11–0.05)	0.527

*Note*: Boldface type indicates statistical significance with two‐sided *p* < 0.05.

Abbreviations: CI, confidential interval; IDI, integrated discrimination improvement; IELSG, International Extranodal Lymphoma Study Group; MSKCC, Memorial Sloan‐Kettering Cancer Center; NRI, net reclassification index; vs, versus.

### 3.7 Webpage development tool

Based on the feasibility of the Radscore‐ILESG model, we further developed a simple‐to‐easy web tool for clinical practice. This application allows users to input Radscore and ILESG values through the interface, after which it calculates the predicted risk of DR to HD‐MTX‐based chemotherapy. The web application is accessible online at https://radscore‐ielsg‐model.shinyapps.io/App‐1/.

## 4 DISCUSSION

In this multicenter cohort study, we investigated the potential predictive capability of prebiopsy T1CE MR image‐based radiomic signature to identify PCNSL patients who were treated solely with HD‐MTX‐based chemotherapy and achieved remission for over 1 year. The proposed radiomic model demonstrated strong performance during external validation. Additionally, we innovatively developed clinical‐radiomic integrated models that exhibited exceptional performance in both the training and validation cohorts. These models show promise as valuable tools for guiding therapeutic decisions at the individual level.

Accurate and individual prediction of DR to MTX‐based chemotherapy is crucial for managing PCNSL patients. However, due to the rarity of this tumor, few studies have focused on this aspect. To the best of our knowledge, this study is the first to develop radiomic‐based models for predicting durable remission in PCNSL patients who solely received HD‐MTX‐based chemotherapy. Given that the extent of therapeutic response evaluated by imaging dose not seems to reflect survival after the end of treatment for PCNSL patients,^25^ our study adopted DR as an endpoint that involves information on both treatment response status and PFS parameter (i.e., posttreatment remission lasting more than 1 year). By using DR, we aim to enhance predictive accuracy in clinical practice. Furthermore, defining DR aligns with scientific rigor by referencing NCCN guidelines, especially given the absence of a consensus on optimal HD‐MTX‐based chemotherapy regimens. Notably, our study boasts relatively large sample sizes (*n* = 105) for radiomic analysis in PCNSL, a rare cancer with an annual incidence of approximately 0.4 per 100,000 in all comers.^26^, ^27^, ^28^, ^29^ Furthermore, unlike previous studies,^16^, ^17^, ^18^ our models were externally validated using data from another institution, enhancing the reliability of our findings.

Our radiomic models were constructed largely based on a homogeneous treatment approach derived from the NCCN guidelines, addressing the challenges posed by treatment regimen variability across institutions and regions.^16^, ^17^, ^18^, ^19^, ^20^ In order to mitigate the predictive bias resulting from additional medications use, our study included more than half of patients administrated HD‐MTX alone during the therapeutic period. It is important to note that this does not imply the additional drugs lack efficacy in the treatment response of PCNSL. Additionally, we observed a significant positive correlation between older age and recurrence risk in the training cohort, but this relationship did not reach statistical significance in the validation cohort. A possible explanation for this discrepancy is the relatively small sample size of the validation cohort, making it challenging to establish a stable relationship between age and PFS. Furthermore, elderly patients often exhibited inferior long‐term tolerance to HD‐MTX treatment. Given the diverse design of HD‐MTX‐based chemotherapy regimens in real clinical practice, it is essential to standardize treatment protocol for this vulnerable population in the future.

To maximize population homogeneity and enhance prediction accuracy, potential confounders significantly associated with defined endpoint have been excluded, such as acceptance of WBRT or ASCT and administration of BTK inhibitors.^30^ Additionally, prebiopsy data were used to analyze MR scans to mitigate postbiopsy bleeding‐related inaccuracies in ROI segmentation. The use of the T1CE sequence for radiomic parameter extraction allowed for the identification of small foci and ensured the stability of manual segmentation in clinical practice, particularly in cases with multiple lesions.

Another contribution of our work is the innovative development of parametric models combining clinical and radiomic signatures for predicting DR to HD‐MTX‐based chemotherapy in PCNSL patients. These integrated models also performed well upon external validation, surpassing the clinical models. This result was expected, given that the two clinical models were initially designed to predict PFS for PCNSL patients, irrespective of the homogeneity of the treatment approach.^19^, ^20^ Therefore, our radiomic signature proves particularly adept at offering precise predictive values for a specific subset of PCNSL patients who have exclusively undergone HD‐MTX‐based chemotherapy. While neither the Radioscore‐IELSG model nor the Radioscore‐MSKCC model significantly outperformed the radiomic signature, DCA showed that the clinical utilization of the Radioscore‐IELSG model was more likely to be stable than that of the other models in both the training and validation cohorts. This suggests that some MR image features may be inherently linked to clinical factors, contributing minimally to the improvement of integrated models' performance. Overall, the results at least indicated the practicality and feasibility of radiomic‐clinical integrated models.

We further developed a user‐friendly online tool based on the Radscore‐ILESG model that have a potential to be translated in clinical practice if adequately validated. Our established online application may offer valuable insights, particularly in identifying patients who are likely to benefit from HD‐MTX‐based chemotherapy. If PCNSL patients are predicted to have high risk of DR to HD‐MTX‐based chemotherapy, considering the administration of such a regimen is advisable. Conversely, for patients predicted to have a low risk of DR, early switching to alternative therapeutic strategies, such as chemotherapy‐free options,^9^ is recommended. Overall, this tool facilitates early decision‐making tailored to individual therapeutic strategies.

There were several limitations in this study. First, potential bias in sample selection and image data acquisition may have occurred due to the retrospective study design. Second, despite the employment of multiple statistical methods to control for confounders, the diversity of HD‐MTX‐based chemotherapy regimens inevitably introduces potential bias in outcome prediction. Furthermore, the heterogeneity of treatment regimens may impact the distribution of patients within the two groups and thus caution in the use of DR/LoDR as the endpoint index should be warranted for this analysis. Therefore, achieving consensus on the standard treatment of PCNSL is crucial to reduce predictive model development error in the future. Third, given the rarity PCNSL, the total number of patients included in our study appears relatively large. However, potential imbalance and biases exist due to the relatively small sample size in the validation cohort (*n* = 29). For example, baseline parameters between the training cohort and validation cohort might have significantly differed if a larger number of patients were enrolled. Moreover, because of the small sample size in the validation cohort, our results may be inconclusive despite adjusting for chemotherapy regimens and other baseline characteristics through multivariable analysis. Forth, this study was conducted in a single region, and the generalizability of the prediction models to other institutions or countries has not been verified. Fifth, although the radiomic signature from the single T1CE sequence provided strong prediction performance, our model may be further improved by integrating multimodality imaging data in the future. Sixth, a fully automated pipeline for tumor segmentation should be applied for PCNSL to reduce the subjective bias from manual processing, which facilitates translation in clinical practice.^31^ Finally, despite the inclusion of a relatively large sample size with independent training and validation cohorts, prospective design and deep‐learning algorithms might be helpful for improving the prediction performance of the model in the future.

## 5 CONCLUSION

In conclusion, we developed and validated a radiomic signature utilizing prebiopsy MR images, with favorable performance in predicting the durable response of PCNSL to HD‐MTX‐based chemotherapy. Our radiomic‐clinical integrated model shows promise for clinical practice, offering potential valuable insights to optimize treatment strategies and guide clinical decision‐making.

## AUTHOR CONTRIBUTIONS

**Haoyi Li:** Conceptualization (equal); formal analysis (equal); investigation (equal); methodology (equal); visualization (equal); writing – original draft (lead). **Mingming Xiong:** Formal analysis (equal); methodology (equal); software (equal); visualization (equal). **Ming Li:** Data curation (equal). **Caixia Sun:** Formal analysis (equal); methodology (equal). **Leilei Yuan:** Data curation (equal). **Qian Chen:** Data curation (equal). **Song Lin:** Supervision (equal). **Zhenyu Liu:** Supervision (equal). **Xiaohui Ren:** Conceptualization (equal); funding acquisition (equal); project administration (equal); resources (equal); supervision (equal).

## FUNDING INFORMATION

This work was supported by Capital Health Research and Development of Special Fund (2022‐2‐1072).

## CONFLICT OF INTEREST STATEMENT

The authors have no conflict of interest.

## ETHICS STATEMENT

This retrospective study was approved by the Institutional Review Board of Beijing Tiantan Hospital (YW2019‐016‐11), and written informed consent for patient was waived.

## Supporting information


Table S1‐S4.

## Data Availability

The data that support the findings of this study are available on request from the corresponding author.
