# Supplementary material for: Radiomic prediction for durable response to high‐dose methotrexate‐based chemotherapy in primary central nervous system lymphoma
Source: Cancer Med. 2024 Sep 10;13(17):e70182. doi: 10.1002/cam4.70182 (PMC11386301; doi:10.1002/cam4.70182)
Supplement: Supplementary file 1 — Table S1‐S4. [file CAM4-13-e70182-s001.docx]

**Supplementary table 1: MRI scanning parameters for the patients**

| **Hospital** | **Scanner** | **No. of patients** | **TR (ms)** | **TE (ms)** | **Matrix** | **Slice Thickness (mm)** | **Slice Gap (mm)** | **Flip Angle** | **PixelSpacing** |
| --- | --- | --- | --- | --- | --- | --- | --- | --- | --- |
| **Centre 1** | GE 1.5T (SIGNA HDe) | 8 | 2379 | 10.50 | [0, 288, 192, 0] | 5 | 6 | 90 | 0.47 |
|  | GE 3T (DISCOVERY MR750) | 24 | 1778 | 19.50 | [0, 320, 192, 0] | 5 | 6 | 111 | 0.47 |
|  | SIEMENS 3T (TrioTim) | 35 | 2000 | 9.80 | [0, 256, 184, 0] | 5 | 6.5 | 150 | 0.43 |
|  | Philips 3T (Ingenia CX) | 9 | 2000 | 20.00 | [0, 308, 215, 0] | 5 | 6 | 90 | 0.45 |
| **Centre 2** | GE 1.5T (GENESIS_SIGNA) | 10 | 360 | 20.00 | [0, 256, 256, 0] | 3 | 3 | 90 | 1.02 |
|  | GE 3T (Signa HDxt) | 11 | 340 | 20.00 | [0, 256, 256, 0] | 3 | 3 | 90 | 1.02 |
|  | Philips 3T (Ingenia CX) | 8 | 6.6 | 3.03 | [0, 240, 240, 0] | 2 | 2 | 8 | 0.47 |

**Supplementary table 2:** **Radiomic features selected by machine learning algorithms**

| **LASSO** | **Ridge** | **ET** | **SVM-RFE** | **RF** |
| --- | --- | --- | --- | --- |
| wavelet_LLH_gldm_DependenceNonUniformityNormalized | wavelet_LLH_firstorder_Variance | wavelet_LLH_glcm_MaximumProbability | wavelet_LLH_gldm_DependenceNonUniformityNormalized | wavelet_LLH_glrlm_GrayLevelNonUniformityNormalized |
| wavelet_LLH_firstorder_InterquartileRange | wavelet_LLH_glrlm_RunPercentage | wavelet_LLH_firstorder_Variance | wavelet_LLH_glcm_Imc2 | log_sigma_4_0_mm_3D_glcm_Imc1 |
| wavelet_LLH_glcm_Imc2 | wavelet_LLH_firstorder_InterquartileRange | original_firstorder_RootMeanSquared | wavelet_LLH_firstorder_Skewness | log_sigma_1_0_mm_3D_firstorder_Skewness |
| wavelet_LLH_glcm_MaximumProbability | wavelet_LLH_firstorder_MeanAbsoluteDeviation | wavelet_LLH_firstorder_10Percentile | log_sigma_3_0_mm_3D_glcm_Idm | wavelet_LLH_firstorder_Skewness |
| log_sigma_3_0_mm_3D_glcm_Idm | wavelet_LLH_glcm_ClusterTendency | log_sigma_1_0_mm_3D_firstorder_Skewness | wavelet_LLH_gldm_GrayLevelVariance | log_sigma_3_0_mm_3D_glcm_InverseVariance |

Abbreviations: ET, extremely randomized trees; LASSO, least absolute shrinkage and selection operator; RF, random forest; SVM-RFE, recursive feature elimination based on a support vector machine

**Supplementary Table 3:** **Baseline comparison between DR and LoDR groups in Training and validation cohorts**

|  | **Training Cohort**  **(n = 76)** | | |  | **Validation Cohort**  **(n = 29)** | | |
| --- | --- | --- | --- | --- | --- | --- | --- |
| **Clinical Characteristics** | **DR**  **(n = 46)** | **LoDR**  **(n = 30)** | **p-value** |  | **DR**  **(n = 19)** | **LoDR**  **(n = 10)** | **p-value** |
| **Age, y, median (IQR)** | 54 (46-60) | 58 (53-65) | **0.044** |  | 58 (45-62) | 57 (54-63) | 0.490 |
| **Gender, n (%)** |  |  | 0.776 |  |  |  | > 0.999 |
| Female | 23 (50.0) | 14 (46.7) |  |  | 9 (47.4) | 4 (40.0) |  |
| Male | 23 (50.0) | 16 (53.3) |  |  | 10 (52.6) | 6 (60.0) |  |
| **KPS Score, median (IQR)** | 80 (70-80) | 60 (50-80) | **0.010** |  | 70 (60-80) | 70 (60-80) | 0.812 |
| **ECOG Score, median (IQR)** | 1 (1-2) | 3 (1-3) | **0.004** |  | 2 (1-3) | 2 (1-3) | 0.788 |
| **IELSG Score, median (IQR)** | 2 (1-2) | 2 (2-3) | **0.045** |  | 2 (2-3) | 2 (2-3) | 0.934 |
| **MSKCC Class** |  |  | **0.004** |  |  |  |  |
| Class I | 15 (32.6) | 4 (13.3) |  |  | 6 (31.6) | 1 (10.0) |  |
| Class II | 24 (52.2) | 11 (36.7) |  |  | 8 (42.1) | 6 (60.0) |  |
| Class III | 7 (15.2) | 15 (50.0) |  |  | 5 (26.3) | 3 (30.0) |  |
| **Involvement of Deep Areas** |  |  | 0.582 |  |  |  | 0.633 |
| Yes | 31 (67.4) | 22 (73.3) |  |  | 16 (84.2) | 7 (70.0) |  |
| No | 15 (32.6) | 8 (26.7) |  |  | 3 (15.8) | 3 (30.0) |  |
| **Multiple Lesions** |  |  | 0.776 |  |  |  | > 0.999 |
| Yes | 23 (50.0) | 16 (53.3) |  |  | 12 (63.2) | 6 (60.0) |  |
| No | 23 (50.0) | 14 (46.7) |  |  | 7 (36.8) | 4 (40.0) |  |
| **Treatment Cycle, median (IQR)** | 10 (8-12) | 5 (3-6) | < **0.001** |  | 9 (8-11) | 7 (6-9) | **0.014** |
| **Treatment Strategy** |  |  |  |  |  |  |  |
| Induction Regimen |  |  | **0.020** |  |  |  | 0.330 |
| MTX alone | 29 (63.0) | 12 (40.0) |  |  | 15 (78.9) | 5 (50.0) |  |
| R-MTX | 2 (4.3) | 1 (3.3) |  |  | 1 (5.3) | 0 (0) |  |
| R-MTX-TMZ-LNDA | 9 (19.6) | 4 (13.3) |  |  | 2 (10.5) | 2 (20.0) |  |
| R-MT | 1 (2.2) | 2 (6.7) |  |  | 0 (0) | 1 (10.0) |  |
| R-MTX-A | 3 (6.5) | 11 (36.7) |  |  | 0 (0) | 1 (10.0) |  |
| R-M-LNDA | 2 (4.3) | 0 (0) |  |  | 1 (5.3) | 0 (0) |  |
| Consolidation Regimen |  |  | < **0.001** |  |  |  | 0.291 |
| MTX alone | 27 (58.7) | 4 (13.3) |  |  | 13 (68.4) | 5 (50.0) |  |
| MTX+other chemotherapy | 2 (4.3) | 2 (6.7) |  |  | 0 (0) | 1 (10.0) |  |
| Other chemotherapy | 16 (34.8) | 9 (30.0) |  |  | 5 (26.3) | 2 (20.0) |  |
| None | 1 (2.2) | 15 (50.0) |  |  | 1 (5.3) | 2 (20.0) |  |
| **Radscore, median (IQR)** | 0.9 (0.9-1.0) | 0.1 (0.0-0.2) | < **0.001** |  | 0.9 (0.6-1.0) | 0 (0.0-0.2) | < **0.001** |

Boldface type indicates statistical significance with two-sided p < 0.05.

Abbreviations: A, Ara-C (Cytarabine); DR, durable response; ECOG, Eastern Cooperative Oncology Group-Performance Status; IELSG, International Extranodal Lymphoma Study Group; IQR, interquartile range; KPS, Karnofsky performance sacle; LNDA, lenalidomide; LoDR, loss of durable response; MSKCC, Memorial Sloan-Kettering Cancer Center; MTX (M), methotrexate; n, number; R, Rituximab; TMZ (T), temozolomide; y, years

**Supplementary Table 4:** **Radiomic features selected for radiomic signature development**

| **Feature name** | **Category** |
| --- | --- |
| wavelet_LLH_gldm_DependenceNonUniformityNormalized | wavelet |
| wavelet_LLH_firstorder_InterquartileRange | wavelet |
| wavelet_LLH_glcm_Imc2 | wavelet |
| wavelet_LLH_glcm_MaximumProbability | wavelet |
| log_sigma_3_0_mm_3D_glcm_Idm | Log |
| wavelet_LLH_firstorder_Variance | wavelet |
| log_sigma_1_0_mm_3D_firstorder_Skewness | Log |
| wavelet_LLH_firstorder_Skewness | wavelet |

Abbreviations: Log, Laplacian of Gaussian
